# Roles of Diet-Associated Gut Microbial Metabolites on Brain Health: Cell-to-Cell Interactions between Gut Bacteria and the Central Nervous System

**DOI:** 10.1016/j.advnut.2023.10.008

**Published:** 2023-10-29

**Authors:** Chong-Su Kim

**Affiliations:** Department of Food and Nutrition, College of Natural Information Sciences, Dongduk Women's University, Seoul 02748, Republic of Korea

**Keywords:** dietary metabolites, gut microbiota, gut–brain axis, astrocytes, microglia, neurons, cell-to-cell interaction, mental health

## Abstract

Gut microbiota have crucial effects on brain function via the gut–brain axis. Growing evidence suggests that this interaction is mediated by signaling molecules derived from dietary components metabolized by the intestinal microbiota. Although recent studies have provided a substantial understanding of the cell-specific effects of gut microbial molecules in gut microbiome–brain research, further validation is needed. This review presents recent findings on gut microbiota-derived dietary metabolites that enter the systemic circulation and influence the cell-to-cell interactions between gut microbes and cells in the central nervous system (CNS), particularly microglia, astrocytes, and neuronal cells, ultimately affecting cognitive function, mood, and behavior. Specifically, this review highlights the roles of metabolites produced by the gut microbiota via dietary component transformation, including short-chain fatty acids, tryptophan metabolites, and bile acid metabolites, in promoting the function and maturation of brain cells and suppressing inflammatory signals in the CNS. We also discuss future directions for gut microbiome–brain research, focusing on diet-induced microbial metabolite-based therapies as possible novel approaches to mental health treatment.

**Statement of significance**

Diet-derived gut microbial metabolites have cell-specific effects on the relationships between gut microbes and brain cells, including microglia, astrocytes, and neuronal cells. Accordingly, approaches to modulate the levels of microbial metabolites have been suggested as a target for treating neuropsychiatric diseases; however, a better understanding of which cells in the brain are affected by bacterial metabolites is needed to develop more tailored treatments.

## Introduction

The human body contains 100 trillion microorganisms, found primarily within the gut, collectively designated the gut microbiota [1]. Research on symbiotic microorganisms in humans has provided considerable evidence of their roles in human health and disease [[1], [2], [3]]. For instance, certain bacteria establish a mutually beneficial relationship with their human host, contributing to physiological maintenance, while others are pathogenic, contributing to disease development and pathogenesis [4]. The loss of healthy microbiota leads to the invasion of pathogenic bacteria, causing myriad conditions ranging from metabolic to neurological diseases [4,5]. Thus, a healthy gut microbiota that maintains an appropriate balance between beneficial and harmful bacteria is important for human health [5].

The functions of the gut microbiota extend beyond the physical borders of the digestive tract [3]. Intestinal microbes constantly communicate with the gut and distal organs of the host [3]. The communication between the gut microbiota and the brain, known as the gut–brain axis, is a bidirectional system in which alterations in gut bacteria can impact the brain and vice versa [3]. This bidirectional communication between the gut microbiota and brain correlates with various neurological and psychological disorders [6], including depression, Alzheimer’s disease, and attention-deficit/hyperactivity disorder [7]. Additionally, recent studies have sought to elucidate the mechanisms associated with the relationship between the gut microbiota and brain [8] by investigating signaling molecules derived from the host and gut microbes [6,8].

The central nervous system (CNS), comprising microglia, astrocytes, and neuronal cells, interacts with gut microbes through signaling molecules, affecting cognition, mood, and behavior [[9], [10], [11]]. This review summarizes the contemporary literature and evidence on the cellular interactions between gut microbes and the brain, specifically focusing on the role of diet-derived metabolites as signaling molecules in cell-to-cell interactions.

### Diet-Derived Microbial Metabolites Linking Gut Microbes to Host CNS Cells

Existing data demonstrate that immune, enteric, and neural pathways mediate communication between commensal bacteria and the CNS [12]. These mechanistic actions appear intertwined and require an in-depth investigation from a systems biology perspective. Although the mechanisms underlying gut–brain communication remain largely unknown, current research suggests that the central feature of this intricate network is instigated by various signaling molecules generated by intestinal microbiota [13].

Microbial signaling molecules directly and indirectly influence the brain [14]. Some gut microbes produce neuroactive molecules, including amino acids and neurotransmitters, in the gut. However, as neurotransmitters (including serotonin, γ-aminobutyric acid, acetylcholine, and noradrenaline) produced by gut bacteria cannot cross the blood–brain barrier (BBB) [15], they indirectly influence the brain by acting on the enteric nervous system [15]. Meanwhile, amino acids (including tyrosine and tryptophan) enter the systemic circulation and can cross the BBB and are utilized as neurotransmitter precursors, directly affecting brain functions [15,16]. Gut bacteria-derived signaling molecules can also facilitate signals to regulate CNS functions by affecting immune responses and inflammation, which is the well-known pathogenesis of psychiatric disorders [17]. Moreover, short-chain fatty acids (SCFAs) produced by gut microbes can modulate the function of peripheral immune cells [17], which is crucial for the homeostasis of brain immunity and neuroinflammation [18]. Collectively, gut microbiota produce various signaling molecules linking the gut–brain axis. This review focuses primarily on the mechanistic actions of diet-derived gut microbial metabolites and their derivatives on brain health.

### Transformation of dietary components by gut microbiota

Gut microbes metabolize dietary components and produce various associated metabolites [19]. Therefore, diet affects the profiles and concentrations of microbially produced metabolites [19]. Microbially produced metabolites in the gut that elicit various physiological responses in the host include SCFAs, tryptophan and its derivatives, as well as bile acid metabolites [19, 20]. Herein, the cell-specific regulation of gut microbiota-produced metabolites derived from dietary components is discussed.

#### SCFAs.

Dietary fibers are metabolized by the gut microbiota in the cecum and colon and are converted to monosaccharides by gut microbiota, which are further fermented, producing SCFAs [21]. The major SCFAs synthesized by gut microbiota include acetate, propionate, and butyrate, accounting for approximately 95% of those produced in the gut [12]. The concentration of each SCFA varies along the gut path, decreasing in the following order: cecum > proximal colon > distal colon [21]. Given that butyrate is primarily used as an energy source for colonocytes, it is present in the peripheral blood at the lowest concentration among the SCFAs [21]. In contrast, propionate and acetate are absorbed through the portal vein [21]. Absorbed propionate is metabolized in the liver by human enzymes, whereas acetate, which is present in the periphery at the highest concentration, travels throughout the body [21]. By acting as signaling molecules through host G protein-coupled receptors expressed in various tissues, SCFAs can affect a wide range of host physiology and pathology, both locally in the gut and systemically in remote cells [21,22].

#### Tryptophan metabolites.

Tryptophan is an essential amino acid metabolized by the host and gut microbiota [19]. The gut microbiota catabolize tryptophan to produce indole and its derivatives, including indole-3-aldehyde and indole-3-propionic acid [23]. These derivatives act as ligands for aryl hydrocarbon receptors (AHRs) [24]. AHR, a ligand-activated transcription factor, regulates immune and inflammatory responses, playing important roles in health and diseases, including neurological diseases [25]. Recent research has highlighted the involvement of AHR in the gut–brain axis, specifically its regulatory impact on CNS glial cells such as astrocytes and microglia.

#### Bile acid metabolites.

Bile acids are synthesized by human and gut microbial enzymes as products of cholesterol metabolism [26]. Cholesterol metabolism first occurs in the liver, where the primary bile acids, such as cholic and chenodeoxycholic acid, are produced from cholesterol [27]. Primary bile acids are conjugated with glycine or taurine in the liver and secreted into the lumen of the intestine in response to dietary intake [26]. Over 95% of the bile acids secreted in the intestine are reabsorbed and recycled back to the liver via enterohepatic circulation; the remaining 5% reach the large intestine to be excreted in feces or are further metabolized by gut microbiota [26,28]. Gut microbiota mediate the deconjugation and dehydroxylation of primary bile acids and produce secondary bile acids, such as ursodeoxycholic acid (UDCA) and tauroursodeoxycholic acid (TUDCA) [26]. These bile acids play various biological roles in facilitating the digestion and absorption of dietary lipids and lipid-soluble vitamins and act as signaling molecules [26]. Circulating bile acids act on multiple receptors, including nuclear (farnesoid X receptor) and cell surface (G protein-coupled bile acid receptor [GPBAR1]). More specifically, bile acid metabolites influence brain function as they can cross the BBB and activate receptors in the brain [26]. Thus, bile acid-mediated gut–brain interactions have received attention for their role in several neurological and psychological disorders, including schizophrenia, Parkinson’s disease, cognitive decline, and depression [26]. However, the cell-specific regulatory roles of bile acid metabolites as signaling molecules in the gut–brain axis remain understudied. Thus, the findings reviewed in this paper regarding the impact of gut microbiota-derived bile acid metabolites on brain cells warrant more extensive investigation.

### Cell-Specific Regulation by Gut Microbial Metabolites

Various cell types exist in the CNS, including neurons, astrocytes, and microglia [29]. In this paper, we review the cell-specific regulatory functions of gut microbiota-produced metabolites in these cells in the CNS.

### Gut microbes and neurons in the CNS

Neurons are generated throughout life in the mammalian brain via neurogenesis through mitochondria-dependent signaling [30]. The reduction of adult neurogenesis in the hippocampus has been linked to memory decline, depression, and anxiety in animals [30]. Recently, metabolites produced by gut microbiota have received considerable attention for their roles in neurogenesis, linking neuronal cells and gut microbes [31]. In fact, microbiota-produced SCFAs, including propionate and butyrate, promote adult neurogenesis by acting on the mitochondria of neural stem cells (NSCs) [31] (Table 1). For instance, according to Ribeiro et al. [31], propionate and butyrate regulate mitochondrial oxidative stress in NSCs by increasing reactive oxygen species (ROS) levels, promoting neurogenic differentiation. More specifically, the levels of early (βIII-tubulin) and late (NeuN) neuronal markers increase at the transcriptional level in NSCs following exposure to propionate or butyrate, suggesting that both SCFAs promote NSC neurogenesis [31]. Additionally, SCFA-induced neurogenesis is regulated by enhanced mitochondrial biogenesis and mitochondrial oxidative stress in NSCs [31]. It is well known that mitochondria contribute to the differentiation of NSCs, and increased mitochondrial ROS levels (mtROS) impact NSC fate [31,32]. Meanwhile, the mitochondrial DNA copy numbers and mRNA expression of mitochondrial transcription factor A (*Tfam*) are increased in NSCs treated with propionate or butyrate, suggesting that both SCFAs also influence mitochondrial biogenesis [31]. The increase in mitochondrial number is mediated in NSCs by preventing the elimination of mitochondrial ROS as propionate or butyrate treatment increases mtROS levels without impacting the abundance of the major ROS scavenger superoxide dismutase 2 or its activator protein NAD-dependent deacetylase Sirtuin-3 [31]. Moreover, SCFAs trigger neuronal differentiation of NSCs via ROS-related and extracellular signal-related protein kinase 1/2 (ERK1/2)-dependent mechanisms [31]. Indeed, increased mtROS levels have been implicated in the suppression of the ERK1/2 pathway, which is correlated with stem cell differentiation [33]. In line with this finding, NSCs treated with propionate or butyrate exhibit reduced levels of phosphorylated ERK1/2, leading to neurogenesis with the elevated expression of βIII-tubulin in NSCs [31]. Collectively, gut microbial metabolite SCFAs, including propionate and butyrate, contribute to the regulation of NSC neurogenesis via the ROS- and ERK1/2-dependent signaling pathways [31].

**TABLE 1:** Gut microbiota-derived metabolites that regulate CNS cells

CNS cell	Microbial metabolite	Model	Dose/duration	Cell-specific effects	Reference
Neuron	SCFAs (propionate and butyrate)	In vitro (NSCs from 14.5-d postcoitum mouse fetal forebrain)	Sodium propionate: 1 mM/24 h incubation Sodium butyrate: 1 mM/24 h incubation	↑ neurogenesis of NSCs (↑*βIII-tubulin* and *NeuN* mRNA levels) ↑ mitochondrial biogenesis of NSCs (↑mtDNA copy number and *Tfam* mRNA levels) ↑ mitochondrial oxidative stress of NSCs (↑mtROS levels, ↔ SIRT3 and total SOD2 protein levels)	Ribeiro et al., 2020 [31]
	Indole	Adult C57BL/6J male mice (10- to 14-wk-old)	Administration of indole-supplemented drinking water: 200 μM indole/5 wk	↑ neurogenesis in the hippocampus (↑CTNNB1, NEUROG2, and VEGF-α mRNA and protein levels) ↑ functional integration of neurons in the hippocampus (↑ SYP and PSD-95 mRNA and protein levels)	Wei et al., 2021 [34]
	Indole-3-propionate	In vitro (human neuroblastoma SH-SY5Y cells)	Treated with conditioned media from indole-3-propionate-treated microglia (indole-3-propionate: 5 μM/24 h incubation)	↑ neuroprotection (↑*Bdnf* and *Ngf* mRNA level)	Kim et al., 2023 [35]
Astrocyte	SCFAs (acetate, propionate, and butyrate)	In vitro (primary astrocytes from postnatal days 1–3 of mouse cortices)	Acetate: 25–1500 μM, propionate: 3.5–35 μM, butyrate: 2.5–25 μM	↓ neuroinflammation (Acetate: ↑*Ahr* and *Gfap* expression in male cortical astrocytes, Propionate: ↑*Il22* expression in male cortical astrocytes) ↑ neuroprotection (Butyrate: ↑*Bdnf* and *Pgc1a* expression in female cortical astrocytes)	Spichak et al., 2021 [36]
	Tryptophan, indole, indoxyl-3-sulfate, indole-3-propionic acid and indole-3-aldehyde	EAE mouse model	Daily oral gavage of indole, indole-3-propionic acid, and indole-3-aldehyde: 400 μg/20 g body weight/14 d, daily intraperitoneal administration of indoxyl-3-sulfate: 200 μg/20 g body weight/14 d	↓ neuroinflammation (↑*Ccl2* and *Nos2* mRNA level in astrocytes of antibiotic-treated EAE mice)	Rothhammer et al., 2016 [37]
	Bile acid metabolites (TUDCA)	In vitro (primary astrocytes from the whole brain of postnatal day 3–5 of mouse)	TUDCA: 70 μM/24 h incubation	↓ neurotoxic polarization of astrocytes (↓ A1 astrocyte-specific gene expression)	Bhargava et al., 2020 [38]
		EAE mouse model	Daily oral gavage of TUDCA: 500 mg/kg body weight/28 d	↓ infiltration of PSMβ8^+^GFAP^+^ cells in the spinal cords of mice with EAE	
Microglia	Serotonin	In vitro (murine microglial BV-2 cells and primary microglia isolated from the hippocampus of embryonic 18 (e18) mouse)	Serotonin creatinine sulfate monohydrate: 25 μM/16 h incubation	↑ Release of microglial exosome (↑ flotillin-1, Alix, and IDE protein level)	Glebov et al., 2015 [39]
	SCFAs (acetate, propionate, and butyrate)	GF mouse	Administration of SCFAs supplemented drinking water: 67.5 mM sodium acetate, 25 mM sodium propionate, and 40 mM sodium butyrate/4 wk	↑ morphology, density, and functional maturity of cortical microglia of GF mice (↑ populations CSFR1^+^, F4/80^+^, CD31^+^ microglia, ↑ number of segments, branching points, terminal points, and cell volume of microglia)	Erny et al., 2015 [40]
	Tryptophan and indoxyl-3-sulfate	EAE mouse model	Administration of diet supplemented with tryptophan: 14 d, daily intraperitoneal injection of indoxyl-3-sulfate: 14 d	↓ neuroinflammation (↓expression of genes involved in NF-κB signaling in microglia, ↓expression of genes associated with EAE pathogenesis in astrocytes)	Rothhammer et al., 2018 [41]
	Indole-3-propionate	In vitro (murine microglial BV-2 cells)	Indole-3-propionate: 1–10 μM/24 h incubation	↓ neuroinflammation (↓IL-1β and TNF-α mRNA and protein levels)	Kim et al., 2023 [35]
	Bile acid metabolites (UDCA and TUDCA)	In vitro (murine microglial BV-2 cells)	UDCA: 300 μg/mL/48 h incubation	↓ neuroinflammation (↓ nitrite production, ↓ expression of the NF-κB-dependent genes)	Joo et al., 2004 [42]
		Animal model of acute neuroinflammation	Intraperitoneal injection of TUDCA: 500 mg/kg body weight, 72 h	↓ neuroinflammation (↓expression of genes involved in the TGF-β pathway in the brain of LPS-treated mice)	Yanguas-Casás et al., 2017 [43]
		In vitro (primary microglia from the whole brain of postnatal days 3–5 of mouse)	TUDCA: 70 μM/18 h incubation	↓ proinflammatory polarization of microglia (↓*Nos2*, *Il1a*, and *Tnfa* mRNA level)	Bhargava et al., 2020 [38]
		EAE mouse model	Daily oral gavage of TUDCA: 500 mg/kg body weight/28 d	↓ infiltration of Mac-2^+^ microglia/macrophages in the spinal cords of mice with EAE	Bhargava et al., 2020 [38]

The upward arrow indicates a statistically significant increase; the downward arrow indicates a statistically significant decrease. The horizontal arrow indicates a statistically nonsignificant change.

Abbreviations: AHR, aryl hydrocarbon receptor; BDNF, brain-derived neurotrophic factor; Ccl2, C motif chemokine ligand 2; CNS, central nervous system; CSFR1, colony-stimulating factor 1 receptor 1; CTNNB1, β-catenin; EAE, experimental autoimmune encephalomyelitis; GF, germ-free; GFAP, glial fibrillary acidic protein; IDE, insulin-degrading enzyme; mtDNA, mitochondrial DNA; mtROS, mitochondrial ROS; NEUROG2, neurogenin 2; NGF, nerve growth factor; NOS2, nitric oxide synthase 2; NSC, neural stem cell; PGC-1, peroxisome proliferator-activated receptor-γ coactivator; PSD-95, postsynaptic density 95; SCFA, short-chain fatty acid; SIRT3, Sirtuin-3; SOD2, superoxide dismutase 2; SYP, synaptophysin; Tfam, mitochondrial transcription factor A; TGF, transforming growth factor; TUDCA, tauroursodeoxycholic acid; UDCA, ursodeoxycholic acid; VEGF-α, vascular endothelial growth factor-alpha.

Indole, a tryptophan metabolite produced by tryptophanase-expressing gut microbes, also serves to regulate the interactions between neurons and gut microbes [34]. An in vivo study indicated that indole has neurogenic effects on the adult mammalian brain [34] (Table 1). Oral administration of indole (200 μM of indole in drinking water for 5 wk) induces neurogenesis by increasing β-catenin (CTNNB1), neurogenin 2 (NEUROG2), and vascular endothelial growth factor-alpha (VEGF-α) at the mRNA and protein levels via the AHR signaling pathway in the hippocampus of adult mice [34]. Indole supplementation also stimulates the functional integration of neurons by increasing the expression of presynaptic synaptophysin and postsynaptic density 95 at the mRNA and protein levels in the hippocampus of mice [34]. In addition, the neurogenic potential of indole has been verified in AHR-knockout (KO) mice (AHR^−/−^) [34]. However, indole supplementation fails to promote neurogenesis as no significant changes occur in the expression of *Ctnnb1*, *Neurog2*, and *Vegfa* in the hippocampus of AHR-KO mice. Thus, dietary tryptophan-derived indole likely exerts its neurogenic effects via the AHR signaling pathway [34], which is closely associated with beneficial cognitive and behavioral outcomes [44]. In addition, indole-3-propionate (IPA) produced by gut microbes has neuroprotective effects through the regulation of microglia–neuron interactions [35]. Neuronal cells cultured with conditioned media from IPA-treated BV-2 microglial cells (5 μM IPA treatment) exhibit significant upregulation of brain-derived neurotrophic factor (*Bdnf*) and nerve growth factor expression, highlighting neuroprotective effects of gut IPA [35].

### Gut microbes and astrocytes in the CNS

Astrocytes are the most abundant cell type in the CNS and are involved in neuronal development, circuit formation, and metabolic support, as well as the protection and repair of the brain from neuroinflammation [45]. Recent studies using in vitro and in vivo mouse models have shown that microbiota-derived SCFAs influence the transcription of genes related to the immunomodulatory and neuroprotective roles of astrocytes in sex-specific responses [36] (Table 1). For example, Spichak et al. [36] studied the effects of physiologically relevant doses of SCFAs on primary cortical astrocyte cultures from male and female mice and reported that the administration of acetate (25–1500 μM) increases *Ahr* and glial fibrillary acidic protein (*Gfap*) gene expression only in male mice. Meanwhile, propionate (3.5–35 μM) increases IL-22 expression only in cortical astrocyte cultures obtained from male mice in a dose-dependent manner [36]. In addition, incubation with butyrate (2.5–25 μM) induces a dose-dependent increase in the mRNA levels of *Bdnf* and peroxisome proliferator-activated receptor-γ coactivator 1a only in cortical astrocyte cultures from female mice [36]. Hence, SCFAs appear to exert sex-dependent effects on the anti-inflammatory and neuroprotective activities of astrocytes [40]. In addition, gut microbial metabolites derived from dietary tryptophan affect astrocytes and suppress CNS inflammation [37] (Table 1).

Gut microbiota depletion via antibiotic treatment is commonly adopted as a research strategy to understand the roles of gut microbiota. In a study conducted by Rothhammer et al. [37], mice with experimental autoimmune encephalomyelitis (EAE; a mouse model of multiple sclerosis) were treated with antibiotics, and the effects of supplementation with bacterial tryptophan catabolites were examined. Dietary supplementation with bacterial tryptophan catabolites, such as indole, indoxyl-3-sulfate, indole-3-propionic acid, and indole-3-aldehyde, reduced neuroinflammation by downregulating the expression of proinflammatory factors, including C-C motif chemokine ligand 2 (*Ccl2*) and nitric oxide synthase 2 (*Nos2*), in astrocytes in an AHR-dependent manner [37] (Table 1).

Additionally, Bhargava et al. [38] reported that the gut bacteria-produced bile acid metabolite TUDCA protects astrocytes from inflammation and neurodegeneration in vitro and in vivo. They also examined the effects of TUDCA in primary astrocyte cultures polarized toward a neurotoxic phenotype (A1: inflammatory phenotype) that secretes toxic factors capable of killing surrounding oligodendrocytes and neurons [46]. Conditioned media from TUDCA-treated astrocytes under proinflammatory stimuli reduced the expression of A1 astrocyte-specific genes involved in interferon (IFN) γ signaling and cytokine signaling (including IFN-inducible GTPase 1, guanine-binding protein 2, and serpin family G member 1 [38] (Table 1). Moreover, daily oral gavage of TUDCA (500 mg/kg body weight for 28 d) reduced the number of PSMβ8^+^GFAP^+^ cells (neurotoxic A1 astrocytes) in the spinal cords of mice with EAE [38]. Taken together, these results indicate that gut bacterial TUDCA could be used as a therapeutic agent for neuroinflammation to target neurotoxic astrocytes specifically. However, additional investigation is required to assess the immunomodulatory roles of astrocytes in the gut–brain interaction, as it remains a misunderstood area of research.

### Gut microbes and microglia in the CNS

Microglia are CNS-resident immune cells that play important roles in various brain diseases, including Parkinson’s and Alzheimer’s diseases, in the context of neuroinflammation, neurogenesis, and synaptogenesis [12]. These primary immune cells in the CNS respond to signals from metabolites produced by gut microbes [47]. One microbial metabolite that influences microglial activity is serotonin, 90% of which is produced from dietary tryptophan in the gut [12]. Treatment of murine microglial BV-2 cells and primary microglia cultures with serotonin increases exosome-associated proteins, such as flotillin-1, Alix, and insulin-degrading enzyme. This effect is blocked by 5-hydroxytryptamine (serotonin) receptor antagonists [39] (Table 1). Considering that exomes—extracellular vesicles released from the fusion of multivesicular bodies with the plasma membrane—participate in cytokine secretion [48], serotonin may be closely associated with the release of cytokine-carrying exomes from microglia [39]. Thus, serotonin-induced microglial exosomes could have important roles in neuroinflammation and related diseases [48].

SCFAs are also involved in regulating microglial homeostasis [40]. This was demonstrated by supplementing the drinking water of germ-free (GF) mice, which lack all gut microbes, with a mixture of 3 SCFAs (acetic acid, propionic acid, and butyric acid) [40]. SCFA treatment normalized abnormalities in cortical microglia morphology, density, and functional maturity [40] (Table 1). In fact, GF mice supplemented with SCFAs exhibited restored microglia maturity, as evidenced by normalized surface expression of colony-stimulating factor 1 receptor 1 (a key regulator of microglial homeostasis) and the mature microglia (F4/80^+^CD31^+^) population [40]. Moreover, SCFA supplementation normalized the number of microglia segments, branching points, terminal points, and cell volume [40].

Additionally, dietary tryptophan and its metabolites, such as indoxyl-3-sulfate, reportedly suppress microglia activation, subsequently dysregulating astrocytes in an EAE animal model [41] (Table 1). That is, EAE mice treated with either tryptophan or indoxyl-3-sulfate exhibit reduced transcript levels of genes involved in NF-κB signaling in microglia [41]. In line with this finding, EAE mice treated with tryptophan or indoxyl-3-sulfate exhibit reduced expression of genes associated with EAE pathogenesis, such as *Ccl2* and *Nos2*, in astrocytes; these effects are blocked in CX3CR1-AHR mice [41]. Hence, dietary tryptophan and its metabolite suppress NF-κB-mediated proinflammatory signals in microglia, associated with astrocyte activation, in an AHR-dependent manner [41]. Collectively, dietary tryptophan and its microbial metabolites have the potential to regulate the interaction between microglia and astrocytes, which is a key mechanism in CNS inflammation. Moreover, in vitro, IPA—an indole derivative produced by gut microbes—protects the microglia from inflammation [35]. IPA-treated microglial BV-2 cells exhibit significant reductions in the concentration of proinflammatory cytokines (IL-1β and TNF-α) following LPS stimulation compared to the LPS-alone treatment group [35].

Bile acid metabolites also elicit anti-inflammatory effects in microglia [49] (Table 1). Joo et al. [42] reported that UDCA, a secondary bile acid produced by gut microbiota, inhibits nitrite production and expression of NF-κB-dependent genes in BV-2 microglial cells under amyloid β peptide stimulus. Similarly, TUDCA contributes to anti-inflammatory effects in microglia [43]. In a mouse model of acute neuroinflammation (mice intravenously injected with bacterial LPS to induce acute neuroinflammation), activation of transforming growth factor (TGF)-β3 immunoreactivity was observed in microglia [43], which is a key factor in the suppression of inflammatory signals [50]. Activation of the TGF-β pathway was further enhanced by intraperitoneal TUDCA treatment [43]. Hence, TUDCA may participate in activating the TGF-β pathway under inflammatory conditions, contributing to its anti-inflammatory effects in microglia [43]. In addition, TUDCA treatment suppresses proinflammatory polarization of microglia in vitro by reducing the expression of *Nos2*, *Il1a*, and *Tnfa* [38] (Table 1). Moreover, TUDCA supplementation (500 mg/kg body weight for 28 d) ameliorates the severity of EAE disease by reducing the infiltration of Mac-2^+^ microglia/macrophages in the spinal cords of mice with EAE through cell-surface GPBAR1 [38]. These results indicate that bile acid metabolites from gut microbiota and their influence on microglia receptors may mediate anti-inflammatory signals in inflammatory neurological and psychological disorders.

### Targeting the Gut Microbial Metabolites in Neuropsychiatric Disorders

Clinical translation of the therapeutic potential of microbial metabolites in neuropsychiatric disease is important in gut microbiome–brain research; however, currently available clinical data is limited. Nevertheless, preclinical studies have reported the roles of microbial metabolites in brain phenotypes and behaviors using animal models of various neuropsychiatric diseases, including autism spectrum disorder (ASD), Alzheimer’s disease, and Parkinson’s disease [51]. For example, in a mouse study using an autism model, supplementation with sodium butyrate ameliorated ASD-like behavior, as indicated by reduced repetitive behavior in a marble burying test [52]. Another study using an Alzheimer’s disease mouse model reported that sodium butyrate treatment improved learning and memory function [53]. Treatment with SCFAs (a mixture of acetate, propionate, and butyrate) improved motor dysfunction—a behavioral characteristic of α-synuclein-overexpressing mice—in a mouse model of Parkinson’s disease [54]. Therefore, approaches to modulate the levels of microbial metabolites have been suggested as a target for treating neuropsychiatric diseases [51]. However, additional evidence from human studies is required to apply microbial metabolites as a therapeutic target in clinical settings.

### Directives for Future Research

**FIGURE 1:**
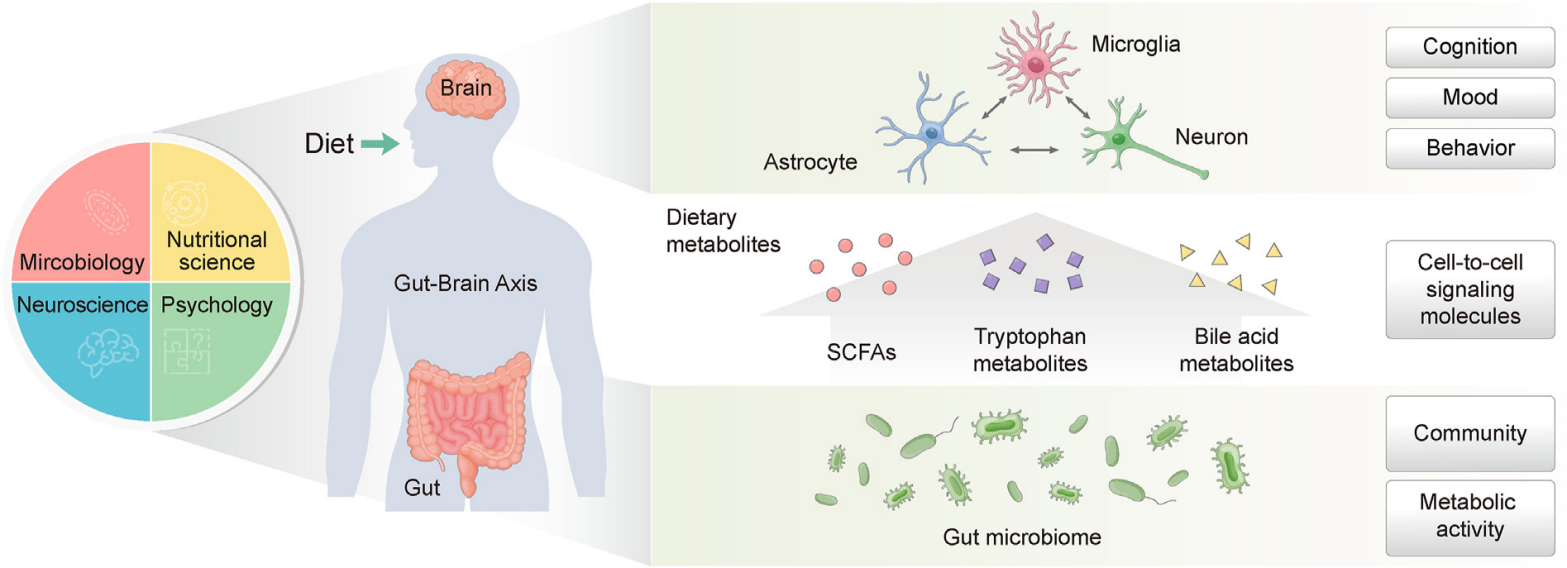
Role of diet-associated metabolites as signaling molecules in cell-to-cell interactions between the gut bacteria and CNS. The gut microbiota have crucial effects on brain function through the gut–brain axis. Gut microbiota-produced dietary metabolites enter the systemic circulation and act as signaling molecules by influencing cell-to-cell interactions between gut microbes and cells in the CNS, particularly microglia, astrocytes, and neuronal cells, which ultimately affect cognitive function, mood, and behavior. Specifically, diet-derived metabolites, including SCFAs, tryptophan metabolites, and bile acid metabolites, promote the function and maturation of brain cells and suppress inflammatory signals in the CNS. To understand the complexity of cell-to-cell interactions between gut bacteria and the CNS, a multidisciplinary dialog between researchers in nutritional science, microbiology, neuroscience, and psychology is necessary. Abbreviations: CNS, central nervous system; SCFA, short-chain fatty acid.

Diet directly affects the metabolite profiles derived from gut microbiota. Dietary interventions focusing on specific signaling molecules that govern the interaction between gut microbes and the brain are promising therapeutic options for treating brain disorders [55,56]. For example, diet and nutrition reportedly regulate the gut–brain axis, which can help alleviate cognitive impairment, anxiety, and depression [[57], [58], [59], [60], [61]] (Figure 1). In fact, compared to prescribed psychiatric drugs, which have adverse side effects, limited treatment periods, and symptom relapse upon treatment cessation, dietary intervention is safe and can function long-term, reducing risk of recurrence [62]. Moreover, empirical data support integrated approaches, combining psychological therapy and nutritional interventions focused on microbial metabolites. These approaches may yield superior outcomes in addressing the intricate gut–brain axis interplay instead of adopting a singular, reductionist approach [63,64].

However, studies on the mechanistic actions underlying the impact of diet on gut–brain interactions are scarce. Hence, future gut microbiome–brain research should focus on characterizing the cell-specific effects of dietary component-derived metabolites on the relationships between microorganisms and brain cells.

Over the past several decades, research methods in gut microbiome science have continued to evolve. In vitro and in vivo preclinical models, including those of GF mice, antibiotic administration, and fecal microbiota transplantation, have been adopted to confirm the validity of gut microbiota-targeted strategies for reducing the symptoms of neurological illnesses and psychological disorders [65,66]. However, the question arises as to what extent in vitro and in vivo rodent models accurately reflect human emotions, cognitive functions, and behaviors, hindering the translation of preclinical findings to humans [65]. Furthermore, evidence suggests that the responses of gut microbiota vary among individuals and are influenced by their baseline gut microbiota composition [67,68]. Meanwhile, nonhuman primate models are genetically similar to humans and have been actively employed to overcome these limitations [69]. In addition, studies have been conducted with small sample sizes and more frequent observations over several time points to confirm the variations in microbial responses in longitudinal studies [70,71]. Nonetheless, a need persists for large cohort studies considering individual gut microbiome-associated factors (genetic characteristics, dietary habits, environmental characteristics, and disease conditions). This approach can facilitate computational modeling analysis to implement personalized microbiome-based therapies targeting brain disorders in clinical settings [72,73]. In line with this approach, intervention studies based on microbial metabolites targeting specific brain cells in clinical trials may significantly advance the research on mental health illnesses.

This review primarily focused on studies that can explain causality in the interaction between the gut microbiota and CNS cells at the cellular level. However, other types of cells in the CNS, including oligodendrocytes and brain endothelial cells, also have important roles in CNS function and neuropsychiatric diseases. A recent study reported that administering a mixture of SCFAs (acetate, propionate, and butyrate) to a rat model of hypoxic–ischemic brain injury suppresses the loss of oligodendrocyte precursor cells by reducing astrocyte activation via the regulation of serum and glucocorticoid-induced protein kinase 1/IL-6 signaling pathway [74]. In addition, butyrate elicits significant effects on the amelioration of demyelination and maturation of oligodendrocyte precursor cells in the organotypic culture of a cerebellar slice [75]. Since research on the gut–brain axis at the cellular level is an emerging area of research, future studies on the impacts of gut microbial metabolites on oligodendrocytes and brain endothelial cells are warranted.

The current review covers select metabolites (SCFAs, tryptophan metabolites, and bile acid metabolites) with well-established evidence of cell-to-cell interactions between gut microbes and brain cells. However, other microbial metabolites have also been implicated in the regulation of gut–brain interactions. For example, trimethylamine N-oxide (TMAO) is a metabolite produced by the gut microbiota from dietary amines that may serve as a risk factor predictor in patients with neurological disorders [76]. Specifically, TMAO administration negatively affects cerebral infarct size and poststroke behavioral outcomes in a mouse model of middle cerebral artery occlusion stroke [77]. Moreover, TMAO treatment of cultured human astrocytes alters the cellular morphology toward activated astrocytes and increases protein markers related to astrocyte activation, such as lipocalin and CD44 [78]. Meanwhile, p-cresol—a tyrosine-derived microbial metabolite—is reportedly a key molecule in several neuropsychiatric disorders, including ASD; however, its mechanistic actions at the cellular level remain unknown [51]. One study reported that cresol treatment impairs oligodendrocyte differentiation, as evidenced by increased expressions of immature progenitor markers in primary cultured oligodendrocyte progenitors [79]. However, further research is needed to demonstrate the mechanistic actions of other microbial metabolites on CNS cells.

A key lesson that has arisen from the past decades of gut microbiome–brain research is that a multidisciplinary dialog between nutritional scientists, biologists, and psychologists is necessary to understand and interpret research outcomes [65,66] (Figure 1). To date, most mainstream gut microbiome–brain research has been conducted by researchers in microbiology [66]. However, understanding the contribution of gut bacteria to dietary metabolism and neurological and psychological outcomes requires greater involvement of nutritionists, neurobiologists, and psychologists [66]. In particular, studies have shown that diet influences gut bacterial enzymes and metabolism [19]. Therefore, an in-depth understanding of the metabolic activities of gut microbiota (Figure 1) by researchers in microbiology and nutritional science is necessary to demonstrate how diet-derived microbial metabolites affect CNS functions.

The microbial ecology of the human body cannot be considered independently from the psychological issues posing considerable burdens on society [80,81]. Recent findings have demonstrated that temperament, personality, and psychopathology are associated with the composition of gut microbes [[82], [83], [84]]. Hence, traits closely related to mental health outcomes and associated social conditions may be corrected by regulating gut dysbiosis. Accordingly, researchers are encouraged to conduct gut microbiome research to inform the development of public policies and systems to address mental health-related social challenges [85].

## Conclusion

A growing body of research indicates that diet-associated gut microbial metabolites regulate the relationship between gut microbes and CNS cells [86]. Therefore, dietary strategies centered on signaling molecules associated with the gut–brain interaction, including supplementation of SCFAs and tryptophan metabolites, are promising therapeutic options for brain disorders [87,88]. However, the mechanistic actions underlying the regulatory effects of dietary metabolites on brain functions require further investigation. Specifically, a more comprehensive understanding is needed regarding which cells in the brain are affected by bacterial metabolites to enable the development of more tailored treatments.

### Author contributions

The sole author was responsible for all aspects of this manuscript.

### Conflict of interest

The author reports no conflicts of interest.

### Funding

This study was funded by by the Technology Innovation Program (No. 20014744) funded by the Ministry of Trade, Industry & Energy (MOTIE, Republic of Korea).

## References

[1] Wekerle H. (2017). Brain autoimmunity and intestinal microbiota: 100 trillion game changers. Trends Immunol.

[2] Almeida A., Mitchell A.L., Boland M., Forster S.C., Gloor G.B., Tarkowska A. (2019). A new genomic blueprint of the human gut microbiota. Nature.

[3] Schroeder B.O., Bäckhed F. (2016). Signals from the gut microbiota to distant organs in physiology and disease. Nat. Med..

[4] Fan Y., Pedersen O. (2021). Gut microbiota in human metabolic health and disease. Nat. Rev. Microbiol..

[5] Cani P.D. (2018). Human gut microbiome: hopes, threats and promises. Gut.

[6] Mayer E.A., Nance K., Chen S. (2022). The gut–brain axis. Annu. Rev. Med..

[7] McGuinness A.J., Davis J.A., Dawson S.L., Loughman A., Collier F., O’Hely M. (2022). A systematic review of gut microbiota composition in observational studies of major depressive disorder, bipolar disorder and schizophrenia. Mol. Psychiatry.

[8] Needham B.D., Kaddurah-Daouk R., Mazmanian S.K. (2020). Gut microbial molecules in behavioural and neurodegenerative conditions. Nat. Rev. Neurosci..

[9] Pereira A., Furlan F.A. (2010). Astrocytes and human cognition: modeling information integration and modulation of neuronal activity. Prog. Neurobiol..

[10] Blank T., Prinz M. (2013). Microglia as modulators of cognition and neuropsychiatric disorders. Glia.

[11] Wu Y., Dissing-Olesen L., MacVicar B.A., Stevens B. (2015). Microglia: dynamic mediators of synapse development and plasticity. Trends Immunol.

[12] Abdel-Haq R., Schlachetzki J.C.M., Glass C.K., Mazmanian S.K. (2019). Microbiome–microglia connections via the gut–brain axis. J. Exp. Med..

[13] Martinez K.B., Leone V., Chang E.B. (2017). Microbial metabolites in health and disease: navigating the unknown in search of function. J. Biol. Chem..

[14] Morais L.H., Schreiber IV H.L., Mazmanian S.K. (2021). The gut microbiota–brain axis in behaviour and brain disorders. Nat. Rev. Microbiol..

[15] Eicher T.P., Mohajeri M.H. (2022). Overlapping mechanisms of action of brain-active bacteria and bacterial metabolites in the pathogenesis of common brain diseases. Nutrients.

[16] Ahmed H., Leyrolle Q., Koistinen V., Kärkkäinen O., Layé S., Delzenne N. (2022). Microbiota-derived metabolites as drivers of gut–brain communication. Gut Microbes.

[17] Dalile B., Van Oudenhove L., Vervliet B., Verbeke K. (2019). The role of short-chain fatty acids in microbiota–gut–brain communication. Nat. Rev. Gastroenterol. Hepatol..

[18] Hoogland I.C., Houbolt C., van Westerloo D.J., van Gool W.A., van de Beek D. (2015). Systemic inflammation and microglial activation: systematic review of animal experiments. J. Neuroinflammation.

[19] Zhang L.S., Davies S.S. (2016). Microbial metabolism of dietary components to bioactive metabolites: opportunities for new therapeutic interventions. Genome Med.

[20] Makki K., Deehan E.C., Walter J., Bäckhed F. (2018). The impact of dietary fiber on gut microbiota in host health and disease. Cell Host Microbe.

[21] Koh A., De Vadder F., Kovatcheva-Datchary P., Bäckhed F. (2016). From dietary fiber to host physiology: short-chain fatty acids as key bacterial metabolites. Cell.

[22] van der Hee B., Wells J.M. (2021). Microbial regulation of host physiology by short-chain fatty acids. Trends Microbiol.

[23] Osadchiy V., Martin C.R., Mayer E.A. (2019). The gut–brain axis and the microbiome: mechanisms and clinical implications. Clin. Gastroenterol. Hepatol..

[24] Quintana F.J., Sherr D.H. (2013). Aryl hydrocarbon receptor control of adaptive immunity. Pharmacol. Rev..

[25] Barroso A., Mahler J.V., Fonseca-Castro P.H., Quintana F.J. (2021). The aryl hydrocarbon receptor and the gut–brain axis. Cell. Mol. Immunol..

[26] Monteiro-Cardoso V.F., Corlianò M., Singaraja R.R. (2021). Bile acids: a communication channel in the gut-brain axis. NeuroMolecular Med.

[27] Winston J.A., Theriot C.M. (2020). Diversification of host bile acids by members of the gut microbiota. Gut Microbes.

[28] Molinero N., Ruiz L., Sánchez B., Margolles A., Delgado S. (2019). Intestinal bacteria interplay with bile and cholesterol metabolism: implications on host physiology. Front. Physiol..

[29] Allen N.J., Lyons D.A. (2018). Glia as architects of central nervous system formation and function. Science.

[30] Zhao C., Deng W., Gage F.H. (2008). Mechanisms and functional implications of adult neurogenesis. Cell.

[31] Ribeiro M.F., Santos A.A., Afonso M.B., Rodrigues P.M., Sá Santos S., Castro R.E. (2020). Diet-dependent gut microbiota impacts on adult neurogenesis through mitochondrial stress modulation. Brain Commun.

[32] Khacho M., Clark A., Svoboda D.S., Azzi J., MacLaurin J.G., Meghaizel C. (2016). Mitochondrial dynamics impacts stem cell identity and fate decisions by regulating a nuclear transcriptional program. Cell Stem Cell.

[33] Na J., Furue M.K., Andrews P.W. (2010). Inhibition of ERK1/2 prevents neural and mesendodermal differentiation and promotes human embryonic stem cell self-renewal. Stem Cell Res.

[34] Wei G.Z., Martin K.A., Xing P.Y., Agrawal R., Whiley L., Wood T.K. (2021). Tryptophan-metabolizing gut microbes regulate adult neurogenesis via the aryl hydrocarbon receptor. Proc. Natl. Acad. Sci. U. S. A..

[35] Kim C.S., Jung S., Hwang G.S., Shin D.M. (2023). Gut microbiota indole-3-propionic acid mediates neuroprotective effect of probiotic consumption in healthy elderly: a randomized, double-blind, placebo-controlled, multicenter trial and in vitro study. Clin. Nutr..

[36] Spichak S., Donoso F., Moloney G.M., Gunnigle E., Brown J.M., Codagnone M. (2021). Microbially-derived short-chain fatty acids impact astrocyte gene expression in a sex-specific manner. Brain Behav. Immun. Health.

[37] Rothhammer V., Mascanfroni I.D., Bunse L., Takenaka M.C., Kenison J.E., Mayo L. (2016). Type I interferons and microbial metabolites of tryptophan modulate astrocyte activity and central nervous system inflammation via the aryl hydrocarbon receptor. Nat. Med..

[38] Bhargava P., Smith M.D., Mische L., Harrington E., Fitzgerald K.C., Martin K. (2020). Bile acid metabolism is altered in multiple sclerosis and supplementation ameliorates neuroinflammation. J. Clin. Invest..

[39] Glebov K., Löchner M., Jabs R., Lau T., Merkel O., Schloss P. (2015). Serotonin stimulates secretion of exosomes from microglia cells. Glia.

[40] Erny D., Hrabě de Angelis A.L., Jaitin D., Wieghofer P., Staszewski O., David E. (2015). Host microbiota constantly control maturation and function of microglia in the CNS. Nat. Neurosci..

[41] Rothhammer V., Borucki D.M., Tjon E.C., Takenaka M.C., Chao C.C., Ardura-Fabregat A. (2018). Microglial control of astrocytes in response to microbial metabolites. Nature.

[42] Joo S.S., Won T.J., Lee D.I. (2004). Potential role of ursodeoxycholic acid in suppression of nuclear factor kappa B in microglial cell line (BV-2). Arch. Pharm. Res..

[43] Yanguas-Casás N., Barreda-Manso M.A., Pérez-Rial S., Nieto-Sampedro M., Romero-Ramírez L. (2017). TGFβ contributes to the anti-inflammatory effects of tauroursodeoxycholic acid on an animal model of acute neuroinflammation. Mol. Neurobiol..

[44] Anacker C., Hen R. (2017). Adult hippocampal neurogenesis and cognitive flexibility—linking memory and mood. Nat. Rev. Neurosci..

[45] Colombo E., Farina C. (2016). Astrocytes: key regulators of neuroinflammation. Trends Immunol.

[46] Liddelow S.A., Guttenplan K.A., Clarke L.E., Bennett F.C., Bohlen C.J., Schirmer L. (2017). Neurotoxic reactive astrocytes are induced by activated microglia. Nature.

[47] Cook J., Prinz M. (2022). Regulation of microglial physiology by the microbiota. Gut Microbes.

[48] Pascual M., Ibáñez F., Guerri C. (2020). Exosomes as mediators of neuron-glia communication in neuroinflammation. Neural Regen. Res..

[49] Yanguas-Casás N., Barreda-Manso M.A., Nieto-Sampedro M., Romero-Ramírez L. (2017). TUDCA: an agonist of the bile acid receptor GPBAR1/TGR5 with anti-inflammatory effects in microglial cells. J. Cell. Physiol..

[50] Li M.O., Wan Y.Y., Sanjabi S., Robertson A.K.L., Flavell R.A. (2006). Transforming growth factor-β regulation of immune responses. Annu. Rev. Immunol..

[51] Swer N.M., Venkidesh B.S., Murali T.S., Mumbrekar K.D. (2023). Gut microbiota-derived metabolites and their importance in neurological disorders. Mol. Biol. Rep..

[52] Kratsman N., Getselter D., Elliott E. (2016). Sodium butyrate attenuates social behavior deficits and modifies the transcription of inhibitory/excitatory genes in the frontal cortex of an autism model. Neuropharmacology.

[53] Govindarajan N., Agis-Balboa R.C., Walter J., Sananbenesi F., Fischer A. (2011). Sodium butyrate improves memory function in an Alzheimer’s disease mouse model when administered at an advanced stage of disease progression. J. Alzheimers Dis..

[54] Sampson T.R., Debelius J.W., Thron T., Janssen S., Shastri G.G., Ilhan Z.E. (2016). Gut microbiota regulate motor deficits and neuroinflammation in a model of Parkinson’s disease. Cell.

[55] Duda-Chodak A., Tarko T., Satora P., Sroka P. (2015). Interaction of dietary compounds, especially polyphenols, with the intestinal microbiota: a review. Eur. J. Nutr..

[56] Rowland I., Gibson G., Heinken A., Scott K., Swann J., Thiele I. (2018). Gut microbiota functions: metabolism of nutrients and other food components. Eur. J. Nutr..

[57] Sun Y., Baptista L.C., Roberts L.M., Jumbo-Lucioni P., McMahon L.L., Buford T.W. (2020). The gut microbiome as a therapeutic target for cognitive impairment. J. Gerontol. A Biol. Sci. Med. Sci..

[58] Kim C.S., Cha L., Sim M., Jung S., Chun W.Y., Baik H.W. (2021). Probiotic supplementation improves cognitive function and mood with changes in gut microbiota in community-dwelling older adults: a randomized, double-blind, placebo-controlled, multicenter trial. J. Gerontol. A Biol. Sci. Med. Sci..

[59] Zmora N., Suez J., Elinav E. (2019). You are what you eat: diet, health and the gut microbiota. Nat. Rev. Gastroenterol. Hepatol..

[60] Shin J.H., Kim C.S., Cha L., Kim S., Lee S., Chae S. (2022). Consumption of 85% cocoa dark chocolate improves mood in association with gut microbial changes in healthy adults: a randomized controlled trial. J. Nutr. Biochem..

[61] Horn J., Mayer D.E., Chen S., Mayer E.A. (2022). Role of diet and its effects on the gut microbiome in the pathophysiology of mental disorders. Transl. Psychiatry.

[62] Muench J., Hamer A.M. (2010). Adverse effects of antipsychotic medications. Am. Fam. Physician.

[63] Clemente-Suárez V.J. (2020). Multidisciplinary intervention in the treatment of mixed anxiety and depression disorder. Physiol. Behav..

[64] Chahwan B., Kwan S., Isik A., van Hemert S., Burke C., Roberts L. (2019). Gut feelings: a randomised, triple-blind, placebo-controlled trial of probiotics for depressive symptoms. J. Affect. Disord..

[65] Cryan J.F., Mazmanian S.K. (2022). Microbiota–brain axis: context and causality. Science.

[66] Sarkar A., Harty S., Lehto S.M., Moeller A.H., Dinan T.G., Dunbar R.I.M. (2018). The microbiome in psychology and cognitive neuroscience. Trends Cogn. Sci..

[67] Kolodziejczyk A.A., Zheng D., Elinav E. (2019). Diet–microbiota interactions and personalized nutrition. Nat. Rev. Microbiol..

[68] Gibbons S.M., Gurry T., Lampe J.W., Chakrabarti A., Dam V., Everard A. (2022). Perspective: leveraging the gut microbiota to predict personalized responses to dietary, prebiotic, and probiotic interventions. Adv. Nutr..

[69] Kuthyar S., Manus M.B., Amato K.R. (2019). Leveraging non-human primates for exploring the social transmission of microbes. Curr. Opin. Microbiol..

[70] Vandeputte D., De Commer L., Tito R.Y., Kathagen G., Sabino J., Vermeire S. (2021). Temporal variability in quantitative human gut microbiome profiles and implications for clinical research. Nat. Commun..

[71] Johnson A.J., Vangay P., Al-Ghalith G.A., Hillmann B.M., Ward T.L., Shields-Cutler R.R. (2019). Daily sampling reveals personalized diet-microbiome associations in humans. Cell Host Microbe.

[72] Asnicar F., Berry S.E., Valdes A.M., Nguyen L.H., Piccinno G., Drew D.A. (2021). Microbiome connections with host metabolism and habitual diet from 1,098 deeply phenotyped individuals. Nat. Med..

[73] Heinken A., Basile A., Hertel J., Thinnes C., Thiele I. (2021). Genome-scale metabolic modeling of the human microbiome in the era of personalized medicine. Annu. Rev. Microbiol..

[74] Gao Y., Xie D., Wang Y., Niu L., Jiang H. (2022). Short-chain fatty acids reduce oligodendrocyte precursor cells loss by inhibiting the activation of astrocytes via the Sgk1/IL-6 signalling pathway. Neurochem. Res..

[75] Chen T., Noto D., Hoshino Y., Mizuno M., Miyake S. (2019). Butyrate suppresses demyelination and enhances remyelination. J. Neuroinflammation.

[76] Praveenraj S.S., Sonali S., Anand N., Tousif H.A., Vichitra C., Kalyan M. (2022). The role of a gut microbial-derived metabolite, trimethylamine N-oxide (TMAO), in neurological disorders. Mol. Neurobiol..

[77] Zhu W., Romano K.A., Li L., Buffa J.A., Sangwan N., Prakash P. (2021). Gut microbes impact stroke severity via the trimethylamine N-oxide pathway. Cell Host Microbe.

[78] Brunt V.E., LaRocca T.J., Bazzoni A.E., Sapinsley Z.J., Miyamoto-Ditmon J., Gioscia-Ryan R.A. (2021). The gut microbiome–derived metabolite trimethylamine N-oxide modulates neuroinflammation and cognitive function with aging. GeroScience.

[79] Gacias M., Gaspari S., Santos P.M.G., Tamburini S., Andrade M., Zhang F. (2016). Microbiota-driven transcriptional changes in prefrontal cortex override genetic differences in social behavior. eLife.

[80] Kim C.S., Shin G.E., Cheong Y., Shin J.H., Shin D.M., Chun W.Y. (2022). Experiencing social exclusion changes gut microbiota composition. Transl. Psychiatry.

[81] Lucas G. (2018). Gut thinking: the gut microbiome and mental health beyond the head. Microb. Ecol. Health Dis..

[82] Sumich A., Heym N., Lenzoni S., Hunter K. (2022). Gut microbiome-brain axis and inflammation in temperament, personality and psychopathology. Curr. Opin. Behav. Sci..

[83] Aatsinki A.K., Lahti L., Uusitupa H.M., Munukka E., Keskitalo A., Nolvi S. (2019). Gut microbiota composition is associated with temperament traits in infants. Brain Behav. Immun..

[84] Kelsey C.M., Prescott S., McCulloch J.A., Trinchieri G., Valladares T.L., Dreisbach C. (2021). Gut microbiota composition is associated with newborn functional brain connectivity and behavioral temperament. Brain Behav. Immun..

[85] Amato K.R., Arrieta M.C., Azad M.B., Bailey M.T., Broussard J.L., Bruggeling C.E. (2021). The human gut microbiome and health inequities. Proc. Natl. Acad. Sci. U. S. A..

[86] Park J., Kim C.H. (2021). Regulation of common neurological disorders by gut microbial metabolites. Exp. Mol. Med..

[87] O’Riordan K.J., Collins M.K., Moloney G.M., Knox E.G., Aburto M.R., Fülling C. (2022). Short chain fatty acids: microbial metabolites for gut-brain axis signalling. Mol. Cell. Endocrinol..

[88] Gao K., Mu C.L., Farzi A., Zhu W.Y. (2020). Tryptophan metabolism: a link between the gut microbiota and brain. Adv. Nutr..

